# Sampling biases obscure the early diversification of the largest living vertebrate group

**DOI:** 10.1098/rspb.2022.0916

**Published:** 2022-10-26

**Authors:** Struan Henderson, Emma M. Dunne, Sam Giles

**Affiliations:** ^1^ School of Geography Earth and Environmental Sciences, University of Birmingham, Birmingham B15 2TT, UK; ^2^ GeoZentrum Nordbayern, Friedrich-Alexander University Erlangen-Nürnberg (FAU), Erlangen, Germany; ^3^ Department of Earth Sciences, Natural History Museum, Cromwell Road, London SW7 5BD, UK

**Keywords:** diversity, sampling standardization, Actinopterygii, fossil record bias, Palaeozoic

## Abstract

Extant ray-finned fishes (Actinopterygii) dominate marine and freshwater environments, yet spatio-temporal diversity dynamics following their origin in the Palaeozoic are poorly understood. Previous studies investigate face-value patterns of richness, with only qualitative assessment of biases acting on the Palaeozoic actinopterygian fossil record. Here, we investigate palaeogeographic trends, reconstruct local richness and apply richness estimation techniques to a recently assembled occurrence database for Palaeozoic ray-finned fishes. We identify substantial fossil record biases, such as geographical bias in sampling centred around Europe and North America. Similarly, estimates of diversity are skewed by extreme unevenness in the occurrence distributions, reflecting historical biases in sampling and taxonomic practices, to the extent that evenness has an overriding effect on diversity estimates. Other than a genuine rise in diversity in the Tournaisian following the end-Devonian mass extinction, diversity estimates for Palaeozoic actinopterygians appear to lack biological signal, are heavily biased and are highly dependent on sampling. Increased sampling of poorly represented regions and expanding sampling beyond the literature to include museum collection data will be critical in obtaining accurate estimates of Palaeozoic actinopterygian diversity. In conjunction, applying diversity estimation techniques to well-sampled regional subsets of the ‘global’ dataset may identify accurate local diversity trends.

## 1. Introduction

There are around 32 000 species of living ray-finned fishes (actinopterygians), amounting to over half of extant vertebrate diversity, and split roughly evenly between marine and freshwater environments [1]. Ray-finned fishes originated in the Palaeozoic, which saw major evolutionary events and changes in the vertebrate fauna, such as the emergence of jaws [2], the rise of actinopterygians [3] and the move onto land [4]. Despite these pivotal changes, and a long history of research on actinopterygians, there are relatively few macroevolutionary studies investigating diversity trends in their early evolution, and all examine face-value patterns of taxonomic richness [3,5–9].

Few studies investigate whether the Palaeozoic ray-fin fossil record is adequate for investigating diversity patterns or whether it is severely biased [3,5,10]*.* Notably, biases may impact the marine and freshwater record differently. For example, trends in Permian freshwater osteichthyan diversity are altered by inclusion of Lagerstätten in analyses, while trends in the marine realm remain consistent with or without Lagerstätten [5]. However, this difference is likely due to the temporal distribution of marine and freshwater Lagerstätten rather than a causal effect related to palaeoenvironment. Despite apparent limitations of the early actinopterygian record, a series of hypotheses have been proposed on the basis of face-value readings of the record. Low taxonomic diversity in the Devonian followed by an explosive increase in the early Carboniferous is generally interpreted as representing a genuine biological signal, on the basis of relative increases in the actinopterygian faunal component [3,10]. In the later Palaeozoic, some authors qualitatively suggest that low Permian diversity is linked to the rarity of suitable deposits [11], while others disagree and attribute the decline in richness among freshwater taxa to the loss of extensive Euramerican freshwater habitats [5]. It has further been proposed that the consistent ecomorphologies in typical Palaeozoic actinopterygians hint at constraints on diversification into new ecologies and habitats and thus low richness [10]. To date, however, previous studies only present face-value counts of actinopterygians through time without employing recent advances in methodologies to estimate diversity trends. An exception to this [5] performed coverage-based rarefaction to compare the Permian and Triassic as a whole, rather than to estimate diversity trends through time.

Assessing the degree to which fossil record biases affect interpretations of richness is critical to obtaining an accurate estimate of diversity trends [12–14]. These biases can be geological [15,16], geographical [17–19] or anthropogenic [20,21] in nature, and recent analyses show that ‘global’ fossil records are intimately linked to the spatial extent of that record [17,18]. Various statistical methods attempt to tease apart bias from genuine changes (e.g. classical rarefaction and residual modelling), though not without complications (e.g. classical rarefaction can flatten diversity patterns [22–25], while residual modelling is dependent on the sampling proxy used and generally unable to determine whether low diversity results from low sampling [26,27]). Recent years have seen the application of coverage-based methods of sampling standardization, such as shareholder quorum subsampling (SQS) and coverage-based rarefaction [25,28], to palaeobiological occurrence databases [17–19,29–33] as a means of deducing trends in palaeodiversity through time. As SQS subsamples intervals to equal levels of completeness, it returns more accurate relative richness estimates between sampled intervals than size-based rarefaction [23], although is still susceptible to some biases [21,24]. Principally, SQS estimates can have a significant evenness signal [21,24,34], which may be particularly important for datasets that are biased in ways that skew the evenness of frequency distributions within sampled intervals. A recently developed richness estimator, squares [35], estimates higher richness when there are numerous rare taxa (i.e. singletons) and when common taxa are especially frequent. Squares is more robust to uneven distributions than SQS, though falls short when the ratio of richness counts to total number of taxa within intervals is very high [24].

Until recently, no comprehensive through-Palaeozoic occurrence database existed [9], with previously published databases limited in scope or not updated [3,5]. Here, we examine modern and palaeogeographic distributions of Palaeozoic actinopterygian occurrences alongside local richness to attempt to tease apart spatio-temporal sampling biases and biogeographic patterns. In addition, we apply coverage-based sampling standardization and squares extrapolation to the newly assembled occurrence database of Palaeozoic actinopterygians to scrutinize patterns of diversity through the Palaeozoic, the suitability of the dataset and the likely extent and impact of sampling biases, allowing assessment of previous hypotheses surrounding Palaeozoic actinopterygian diversity.

## 2. Methods

### (a) Data preparation

Global occurrences of Palaeozoic Actinopterygii [9], incorporating information on stratigraphy, lithology, geography, age and taxonomy compiled from the literature, were screened for taxonomically indeterminate occurrences. Scale- and teeth-based occurrences were also removed as it is often difficult to assign them definitively to a taxon. Well-preserved fossils are generally required to attribute osteichthyan fossils to a genus [5], and it is difficult to assign isolated scales from the same deposits to different species rather than distinct morphotypes from different parts of the body of one taxon [36,37] unless the isolated scales are directly comparable with contemporaneous body fossils (e.g. Gneudna and Gogo [38]). ICS stratigraphic stages of each occurrence were replaced with intervals of roughly equal length (approx. 9 Ma), determined by either combining shorter intervals (e.g. Kasimovian [3.3 Ma] and Gzhelian [4.8 Ma] = Kasimovian and Gzhelian [8.1 Ma]), or splitting longer intervals (e.g. Visean [15.8 Ma] = early Visean [Chadian-Holkerian; 8.7 Ma] and late Visean [Asbian-Brigantian; 7.1 Ma]; boundary based on the age of the Dunsapie basalt, see [39]).

After the removal of indeterminate occurrences and occurrences that could not be assigned to equal-length intervals, this resulted in a dataset of 1611 occurrences of 473 species (belonging to 226 genera), from 512 unique geographical localities. Generally, unique geographical localities represent unique assemblages in time and space and can therefore be considered communities for the purposes of local richness. However, a limited number (usually the more productive and explored localities) may not be unique in space or time for one of two reasons. First, multiple horizons may outcrop at a single geographical locality, for example at Glencartholm, where different taxa are found in separate horizons (e.g. *Mesopoma*: Horizon K; *Rhadinichthys*: Horizon F). Here, we treat Glencartholm as a single locality due to the occurrence of most fossils at the Macconochie site which has uncertain stratigraphy [40]. Second, the same horizon may outcrop at multiple localities, for example at Bear Gulch, where multiple locations are known within a confined local area [41]. Here, we treat these separate outcrops as separate localities with consistent stratigraphic information.

The cleaned dataset was then used for local richness and diversity estimation. Local richness estimates included occurrences that could not be constrained to a single interval by using the midpoint of their range as their occurrence age, while only occurrences attributable to a single interval were used in diversity estimation. All analyses were conducted within R v.4.1.0 [42].

### (b) Alpha diversity (local richness)

Species per locality were counted as a measure of alpha diversity (local richness [43]). Occurrences indeterminate at species level were retained in line with the taxonomic hierarchy of the Paleobiology Database. Modern coordinates for these localities were translated into palaeocoordinates using the R ‘chronosphere’ package (v.0.1.5 [44]). Local richness was then subset by marine and freshwater environment (brackish environments were included in marine counts) and plotted against palaeolatitude. Additionally, palaeogeographic maps showing local richness were produced in ‘chronosphere’ [44] for each interval. It is uncertain whether some Permian localities (Pastos Bons—Brazil; Deep Red Run, Dundee, McCann Quarry, Pond Creek, South Dakota State Cement Plant Quarry—USA; Sobernheim—Germany) are Artinskian or Kungurian in age, and these localities are therefore plotted in both palaeogeographic maps.

### (c) Sampling standardization and diversity estimation

Coverage-based sampling standardization [22,28,45,46] was implemented to estimate global diversity patterns, first using coverage-based rarefaction, which is mathematically very similar to SQS, via the R package iNEXT (v.2.0.19 [47]), following the procedure outlined in Dunne *et al.* [30]. The data were rarefied by geographical locality by analysing incidence-frequency matrices of the occurrence data. Extrapolated estimates were limited to no more than twice the observed sample size [47]. Coverage-rarefied richness was computed at genus level using roughly equal-length bins, at quorum levels 0.3–0.7; higher quorums were unattainable and resulted in estimates exceeding double the reference sample size. Devonian bins were excluded due to the very small sample sizes and low levels of coverage. Coverage in the early Visean and Kungurian is also extremely low, and estimates for these intervals will therefore be unreliable, particularly at higher quorums. Rank occurrence distributions and size- and coverage-based rarefaction curves were generated for each interval to investigate the reliability of coverage-rarefied richness estimates. A split-sum test was also conducted after dividing the dataset into marine (including brackish) and freshwater occurrences, although the resultant low sample sizes meant that the highest attainable quorum was very low.

A second sampling standardization method, squares, was also implemented (following Allen *et al.* [32]) due to the susceptibility of coverage-rarefied richness to unevenness in the underlying distribution of the data [23]. Squares, unlike other extrapolators such as Chao 1, is designed to minimize underestimation resulting from unevenness (i.e. when there are lots of singletons and common taxa are extremely frequent) [35]. Squares is also more accurate than other extrapolators (e.g. *λ*_5_) and performs well in split-and-sum tests [24]. Squares-extrapolated estimates of genus and species richness were conducted in R by applying Alroy's equation [35], following the same procedure as Allen *et al.* [32]. In addition, we conducted split-and-sum tests after dividing the dataset into marine (including brackish) and freshwater occurrences.

## 3. Results

### (a) Alpha diversity (local richness)

**Figure 1.**
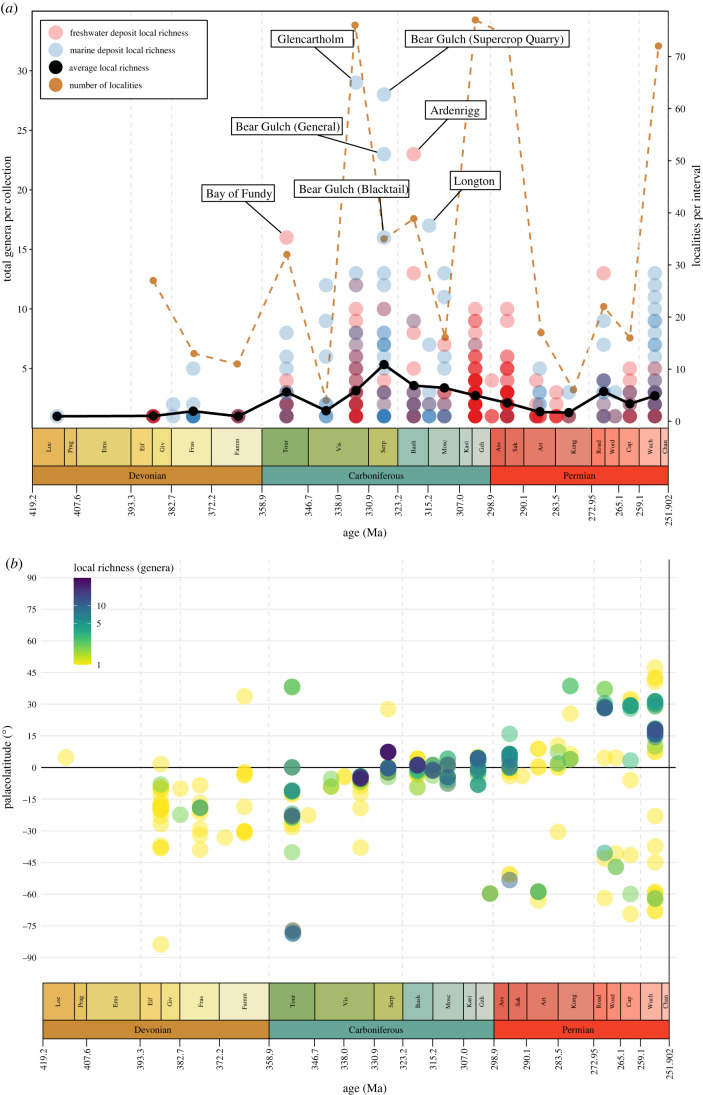
Local richness (number of species per fossil locality) of actinopterygians through the Palaeozoic. (*a*) Local richness plotted by environment, separated by freshwater (red) and marine (blue; incorporates brackish occurrences). Colour saturation (transparency) indicates density of localities, and the most species-rich localities are labelled. Note that purple indicates contemporary localities with a similar diversity of marine and freshwater actinopterygians. (*b*) Palaeolatitude of localities through time, with local richness indicated by colour (yellow localities have low richness, progressing through green to the most diverse localities in indigo). (Online version in colour.)

Local richness is generally low in the Devonian (figure 1), with only one locality containing more than three genera (Paddy's Valley, Gogo Formation, Frasnian, Australia). Levels of local richness are highest in the Carboniferous, particularly around the Serpukhovian-Bashkirian boundary (figure 1), before declining steadily in the latest Carboniferous (Kasimovian and Gzhelian) and early Permian (Cisuralian). Notable localities contributing to the mid-Carboniferous peak include Glencartholm (Scotland, late Visean, marine), Ardenrigg (Scotland, Bashkirian, freshwater), Longton (England, Bashkirian, marine) and the Bear Gulch localities (USA, Serpukhovian, marine) (figure 1*a*). With the exception of two notable localities (Kinney Brick Quarry, US, and Rio Negro, Uruguay) there are very few occurrences with marine and brackish palaeoenvironments in the latest Carboniferous (Kasimovian and Gzhelian) and earliest Permian (Asselian and Sakmarian). Instead, the vast majority of occurrences are freshwater in origin. However, few freshwater localities are present in the Artinskian and Kungurian, which yield very low richness, and occurrences and richness from marine settings also remain low. In the latest Permian (Wuchiapingian and Changhsingian), marine localities generally have much higher genus counts than freshwater localities. Broadly, it is clear that the sampling of different palaeoenvironments throughout the Palaeozoic is highly heterogenous, with many intervals showing a clear dominance of either marine or freshwater occurrences (figure 1; electronic supplementary material, figure S1).

### (b) Palaeomaps and geographical spread

#### (i) Devonian

**Figure 2.**
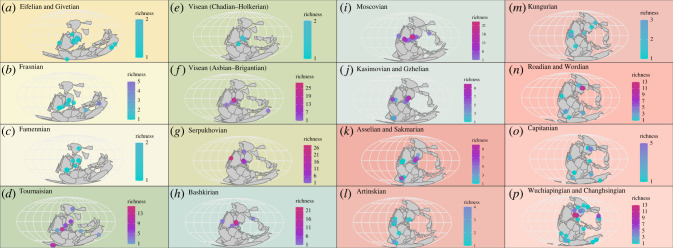
Palaeomaps showing palaeogeographic spread and local richness of individual localities through time plotted in roughly even-length intervals. Colour scales correspond to the richness of localities, ranging from low (light blue) to high (pink) richness. (*a*) Eifelian & Givetian; (*b*) Frasnian; (*c*) Famennian; (*d*) Tournaisian; (*e*) Visean (Chadian−Holkerian); (*f*) Visean (Asbian−Brigantian); (*g*) Serpukhovian; (*h*) Bashkirian; (*i*) Moscovian; (*j*) Kasimovian & Gzhelian; (*k*) Asselian & Sakmarian; (*l*) Artinskian; (*m*) Kungurian; (*n*) Roadian & Wordian; (*o*) Capitanian; (*p*)Wuchiapingian & Changhsingian. (Online version in colour.)

Despite their earliest occurrence being just north of the palaeoequator (*Meemannia*, Lochkovian, South China), actinopterygians are known almost exclusively from southern palaeolatitudes in the Devonian (figures 1*b* and 2*a*). Only two other Northern Hemisphere occurrences are reported (*Cheirolepis*, Givetian, Svalbard [48]; *Krasnoyarichthys*, Famennian, Russia [49]). The majority of taxa occur at low palaeolatitudes (0° to −30°), with a small number just crossing into the mid-palaeolatitudinal band (−30° to −60°). A clear outlier, near the southern palaeopole (−83.81°), is the recently reported *Austelliscus ferox* from Brazil [50].

Devonian actinopterygian occurrences mirror both continental configurations—the majority of landmasses and shallow seas were palaeoequatorial and in the Southern Hemisphere [51]—and the broader Devonian fossil record [18,52,53]. Givetian and Eifelian occurrences are dominated by European (especially Scottish) deposits, with limited contributions from the USA, Australia, the Antarctic and Brazil (figure 2*a*). By contrast, Frasnian occurrences (figure 2*b*) are dominated by the Australian Gogo Formation, with fewer occurrences from Europe and North America, and a single occurrence from Iran. The USA dominates Famennian occurrences (figure 2*c*), with additional occurrences from Russia, Greenland and Belgium.

#### (ii) Carboniferous

In general, Carboniferous localities have both higher local richness and a broader palaeolatitudinal spread than in the Devonian, although are generally still restricted to low and southern palaeolatitudes (figure 1*b*). Most Tournaisian localities are clustered at low palaeolatitudes around the southern edge of Euramerica in regions that correspond to present-day Canada, USA, UK and European Russia, although the Waaipoort Formation in South Africa (−78°) represents the richest high-palaeolatitude locality of the entire Palaeozoic. Localities with lower local richness are found in Australia, Turkey and Siberia. By contrast, early Visean (Chadian-Holkerian) low palaeolatitudes are few in number and extremely depauperate (figure 2*e*).

For much of the rest of the Carboniferous, local richness greatly increases while palaeolatitudinal spread decreases. Other than single occurrences from Australia and the USA, all late Visean actinopterygians are clustered in the UK and Ireland, including the highly diverse Glencartholm locality (figures 1*b* and 2*f*). Similarly, in the Serpukhovian (figure 2*g*), only a single occurrence is found outside a 20° palaeolatitudinal band centred around the palaeoequator encompassing UK localities, a single Belgian locality and the speciose Bear Gulch localities. Geographical spread continues to decline in the Bashkirian (figure 2*h*) and Moscovian (figure 2*i*), with all but one occurrence within 10° of latitude of the palaeoequator. Again, localities are primarily known from Europe (Belgium, Czechia, France, Ireland, UK) and North America (Canada, USA), with a single Bashkirian taxon known from a low-latitude locality in the eastern Palaeotethys (China). The only latest Carboniferous (Kasimovian and Gzhelian; figure 2*j*) locality outside of this band is the −60° Gzhelian Ganigobis Shale, which outcrops in South Africa and Namibia, albeit with low local richness. Broadly, Carboniferous actinopterygian palaeolatitudinal distribution matches other contemporaneous groups [18,53].

#### (iii) Permian

Compared to the Carboniferous and Devonian, Permian occurrences generally display a broader geographical spread (reflecting increases in the broader fossil record [18,53]) but lower local richness. The extent of palaeogeographic sampling in the Asselian and Sakmarian (figure 2*k*) is greater than the Kasimovian and Gzhelian, with more occurrences at higher palaeolatitudes, including the diverse Uruguayan fauna from Rio Negro (−53°). The Artinskian (figure 2*l*) is the most depauperate interval of the Permian, despite a comparatively high palaeogeographic spread: the locality with the highest local richness, Loeriesfontein, contains only four genera. Contrary to most other Palaeozoic intervals, there are very few European Artinskian localities.

From the Kungurian (figure 2*m*) onwards, localities occur across the broadest palaeolatitudinal spread of the entire Palaeozoic. This includes the first sampling of northern mid-palaeolatitudes since the Tournaisian. Roadian and Wordian localities (figure 2*n*) with the highest local richness are found in Russia, centred around 30° palaeolatitude, although less diverse occurrences are seen at high southern palaeolatitudes in Brazil, India and Zimbabwe. In contrast with most other intervals, only two depauperate localities occur near the palaeoequator. This trend continues into the Capitanian (figure 2*o*), where localities yielding few genera are found across a wide range of palaeolatitudes, with very few at equatorial latitudes, and most diversity stems from Russia.

The Wuchiapingian and Changhsingian interval (figure 2*p*) has the broadest geographical spread in sampling of the Palaeozoic, possibly due to intensive research focus on the Permo-Triassic mass extinction event [54,55]. Numerous localities are spread from southern mid- to high-palaeolatitudes, including opposing sides of the palaeopole (present-day South Africa and Australia). Notably, this interval contains the first Permian actinopterygians from the eastern Palaeotethys (present-day China). Northern low- to mid-palaeolatitudes have the highest local richness, stemming from assemblages in the UK and Germany, Russia and Greenland.

### (c) Palaeodiversity estimates

#### (i) Coverage-based rarefaction

**Figure 3.**
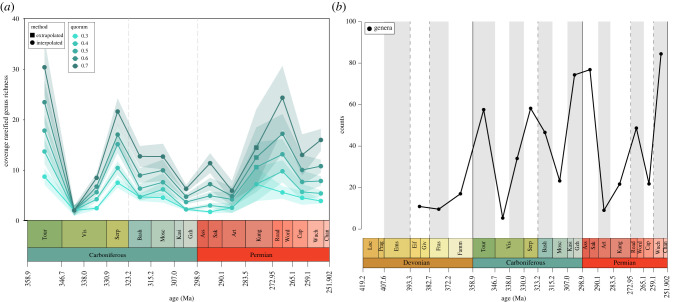
(*a*) Coverage-based rarefaction estimates of Carboniferous and Permian actinopterygian diversity at genus level, showing estimates for different quorum levels in different colours from low quorums (0.3) in light blue to higher quorums (0.7) in darker blue. The shaded areas for each quorum are confidence intervals of estimates. Devonian intervals were removed (see Methods). Estimates were either interpolated (circles) or extrapolated (squares) up to twice the reference sample size [47]. (*b*) Squares diversity estimates of Devonian to Permian genus richness, plotted at the midpoints of equal-length intervals. (Online version in colour.)

Estimates of relative genus richness using coverage-based rarefaction (figure 3*a*; for species-level trends, see electronic supplementary material, figure S2*a*) suggest an overall decline in diversity through the Carboniferous, with a sharp rise then subsequent fall in the Permian. Richness levels decrease rapidly from a peak in the Tournaisian to the early Visean with a small increase in the late Visean, before rising sharply in the Serpukhovian. The remainder of the Carboniferous is marked by a general decline, with the lowest observed values in the Kasimovian and Gzhelian, another intensely sampled interval. Richness estimates rise slightly across the Carboniferous-Permian boundary, with a drop in the Artinskian followed by a steady rise through the Kungurian to the Roadian and Wordian. A decline marks the remainder of the Permian at low quorums, though there is a slight increase in the latest Permian at higher quorums.

#### (ii) Squares

Squares diversity estimates contrast with coverage-based rarefaction estimates: where coverage-based rarefaction returns low estimates, squares estimates are generally high. Squares-extrapolated genus richness estimates (figure 3*b*; for species-level estimates see electronic supplementary material, figure S2*b*) gradually increase throughout the Devonian and into the Tournaisian. Early Visean estimates drop sharply, before gradually rising in the late Visean to Serpukhovian. A slight decrease into the Bashkirian is followed by a steeper decline in the Moscovian. The highest estimates thus far are seen in the latest Carboniferous with a further increase into the Asselian and Sakmarian, followed by a precipitous drop in the Artinskian. Richness estimates rise in the Kungurian and marginally in the Roadian and Wordian before dropping in the Capitanian. The latest Permian (Wuchiapingian and Changhsingian) is the most diverse interval of the Palaeozoic.

#### (iii) Split-and-sum tests

Split-and-sum squares estimates for marine and freshwater subsets generally return greater estimates than the complete dataset (electronic supplementary material, figure S3*a*), though there is no systematic trend related to the dominance of occurrences of a particular palaeoenvironment. Rather, estimates deviate more (i.e. are higher) from the total dataset when occurrences are dominated by either palaeoenvironment. This occurs because the squares equation estimates much higher diversity when there are more rare taxa, and therefore intervals dominated by marine occurrences tend to have much higher extrapolated estimates for freshwater diversity in the split datasets, and vice versa. Regardless, subsetting the dataset by palaeoenvironment leads to extremely poor sample sizes for either palaeoenvironment in most intervals. Average sampling probability in each interval was similar between environmental subsets (marine – 0.55, freshwater – 0.57) indicating no systematic bias in sampling of palaeoenvironments; however, there is great variability in the sampling probability through time. Coverage-rarefaction split-and-sum tests resulted in very large deviations (electronic supplementary material, figure S3*b*) due to small samples and very low coverage, giving no meaningful information relative to the total dataset analysis.

## 4. Discussion

### (a) Biogeographic trends and biases

Palaeozoic actinopterygian occurrences are overwhelmingly geographically biased towards the Northern Hemisphere: fewer than 9% of known localities (52/586) are from the Southern Hemisphere [9]. Certain regions are notably underrepresented throughout the Palaeozoic, such as the northern, eastern and southern Palaeotethys (present-day Middle East, south and east Asia, north Africa) and the northern coastline of Laurussia (present-day Siberia, Kazakhstan and interior of Asia). Sampling through much of the Devonian and Carboniferous is limited to a narrow band around the palaeoequator, largely corresponding to present-day Europe and North America (figure 2), which also contain localities with the highest local richness [9]. The most diverse localities trend from low- to mid-palaeolatitudes through the Palaeozoic, essentially tracking the migration of North America and Europe (figure 1*b*). Reporting new taxa from underrepresented regions [48,50] will have major implications for palaeogeographical spread, patterns of diversity and interpretations of ray-finned fish evolution, especially in the face of taxonomic revisions invalidating many existing generic referrals [56,57].

Ideally, rarefaction curves for sampled intervals should be close to asymptote before performing diversity estimation techniques to ensure that future sampling will not drastically alter face-value counts of richness. Inspection of the Palaeozoic ray-finned fish record suggests this condition has not yet been reached (electronic supplementary material, figure S4). In the short term, increased sampling of the most undersampled intervals will improve comparability. However, research focus on taxa from well-sampled regions that remain undescribed in museum collections [58,59] is also vital for attaining accurate estimates of actinopterygian diversity in the Palaeozoic, particularly at local scales. For example, indeterminate actinopterygians currently account for over 50% of fish fossils at the Kinney Brick Quarry locality [60].

Both marine and freshwater deposits are recorded throughout the Palaeozoic, with the number of sampled marine and freshwater deposits roughly tracking each other through much of the Carboniferous. However, marine palaeoenvironments are scarce in the later Palaeozoic. This long-recognized Permian scarcity [10,11,61] also extends back into the late Carboniferous (figure 1*a*; electronic supplementary material, figure S1). The near-complete lack of marine deposits bearing actinopterygians and the prevalence of freshwater deposits is also reflected in PBDB-derived gnathostome occurrences (electronic supplementary material, figure S5), and likely tracks the relative absence of marine deposits in western Europe [62]. Marine global rock volume reduces from the Carboniferous into the Permian, though is still higher than non-marine [63]. Indeed, marine deposits are common outside of Europe (e.g. North America [64]), and geographical plots of marine gnathostome occurrences from the PBDB (electronic supplementary material, figure S6) show significant Permian diversity outside of Europe.

Considered together, this may indicate that a regional decrease in the availability of deposits is partially responsible for the apparent reduction in actinopterygian diversity in the early Permian, but that there is a genuine global decline relative to other gnathostomes. However, close inspection of marine gnathostome occurrence records show them to comprise taxa which are far more readily identifiable on the basis of limited material (e.g. sharks [65]), suggesting that the actinopterygian record is affected by a taphonomic bias. The dominance of European occurrences in the actinopterygian dataset suggests that low marine diversity in this period is linked to a sampling bias and regional absence of these rocks rather than a true biological signal. There is certainly a change in the sampling of terrestrial vertebrates from aquatic to dryland terrestrial environments across the Carboniferous-Permian [66], and a similar change may explain the drop in sampling of Permian actinopterygians. Concurrent with this environmental shift is a noticeable palaeogeographical expansion: rather than being restricted to palaeoequatorial regions, Permian occurrences are reported further from the palaeoequator, reflecting the distribution of exceptional localities in the fossil record [63]. It is unclear to what extent this represents a shift in sampling regime rather than an ecological expansion, though it may mirror the reduction in sampling of Euamerican deposits [66].

### (b) Palaeozoic actinopterygian diversity patterns

Broadly, it appears that the actinopterygian fossil record is too poor to deduce accurate diversity patterns through most of the Palaeozoic, and we caution against interpreting face-value patterns. Past works have interpreted the actinopterygian record as a general post-extinction recovery curve, with moderate diversification in the early Tournaisian, a peak in the mid-late Visean, and stability in the late Mississippian onwards [3,10,67]. In contrast, both our analyses recover an initial peak in the Tournaisian, perhaps reflecting high initial diversification as actinopterygians replaced other extinct gnathostomes [3,10,68]. The sharp drop into the Visean results from the use of equal-length bins: the early Visean is extremely poorly sampled, and although the late Visean is the most intensely sampled interval of the Carboniferous, diversity estimates remain comparatively low. No prior study has investigated the Pennsylvanian below epoch level, but trends in the Permian broadly agree with past work [5]. Heightened sampling may be responsible for the apparent early Permian peak in face-value actinopterygian richness.

Changes in mean local richness largely track changes in ‘global’ (gamma) raw diversity [9], with the exception of the latest Carboniferous and earliest Permian (figure 1). In the late Carboniferous and early Permian, high levels of sampling (localities and equal-area grid cells [9]) of isolated localities with low alpha diversity drive high ‘global’ diversity, with few contributions from diverse assemblages (figure 1). These richness patterns are drastically different to those reported for Palaeozoic tetrapods [30], and the overall decrease from the Carboniferous to Permian contrasts the biodiversification of invertebrates over the same period [69].

In contrast with coverage-rarefied richness estimates, extrapolated estimates from squares analysis return very similar trends to face-value counts of richness [3,5,9]. These differences persist regardless of whether sampling is via equal-length intervals or geological stages and are likely due to taxonomic biases (see below). This recalls recent work on Palaeozoic tetrapods, which found that diversity patterns among reptiles and synapsids changed significantly depending on the quorum levels or use of squares [70]. For example, coverage-rarefied actinopterygian richness decreases from the Tournaisian peak to the late Visean in contrast with previous hypotheses [3,6,9], yet both the face-value counts and squares estimates of species increase significantly from the Tournaisian to late Visean. There is consensus, however, in the high diversity of the Serpukhovian [3,9], indicating genuine diversity, though the vast majority of this is driven by the diverse Bear Gulch fauna.

Trends into the Pennsylvanian also differ, with the greatest difference seen in diametrically opposed estimates for the Kasimovian and Gzhelian, which is attributable to how the methods estimate diversity. The same is also true for the Asselian and Sakmarian and late Permian. Coverage-rarefied richness estimates depend on the attainable level of coverage, and examination of occurrence distributions (electronic supplementary material, figure S7) and rarefaction curves (electronic supplementary material, figures S8 and S9) reveals that at higher coverage, the Kasimovian and Gzhelian would most likely represent one of the most diverse intervals. Squares, however, estimates higher richness when there are many singletons and when common taxa are especially common [24], and these intervals fulfil both of these criteria. The combined presence of high-frequency taxa and numerous singletons results in these conflicting estimations for some intervals.

Taxonomy also plays a key role. The observed rise in early Permian species-level diversity estimates in both analyses and face-value readings [9] reflects the presence of numerous species of few genera (namely *Amblypterus* and *Paramblypterus*). The problem of high-frequency genera is not unique to actinopterygians; such genera are known to bias other osteichthyan groups [71]. By contrast, Kungurian estimates are based on very few occurrences of monospecific genera, and sampling of a high number of genera at low quorums results in high—yet unreliable—genus-level coverage-rarefied richness estimates. The extremely high Roadian and Wordian estimates in the analyses can also be explained by high numbers of singletons and relative absence of common genera.

### (c) Unevenness in the actinopterygian fossil record

Coverage-based rarefaction techniques produce the most reliable richness estimates when rank occurrence does not differ considerably between samples, even when samples have comparable face-value richness [22–25,28,34,45]. Unevenness in occurrence distributions can therefore heavily influence the reliability of diversity estimates. Rank occurrence distribution plots for Palaeozoic actinopterygian genera and species indicate extreme unevenness within intervals and variation in evenness between intervals (electronic supplementary material, figure S7). Some intervals (e.g. Kasimovian and Gzhelian) contain one or two taxa with more than 60 occurrences, a handful with between 30 and 10 occurrences, and a long tail of singletons or doubletons; others (e.g. Tournaisian) have a more even distribution. Differences can even arise between the genus- and species-level occurrence distributions in the same interval: in the Asselian and Sakmarian most species-level diversity stems from multiple species of two genera, resulting in low genus estimates at lower quorums, but higher species-level estimates due to the more even occurrence distributions (compare electronic supplementary material, figures S3*a*,*b*, S4*c* and S5*c*).

Much of this imbalance is driven by ‘waste-basket’ genera erected by monographic descriptions [72–75], despite a wide range of varied morphologies and extensive temporal and geographical ranges within genera [9,76,77]. Palaeozoic taxa typically lack characters that can be easily attributed from isolated remains and rely on well-preserved material in order to be confidently identified. Historical practice often relied on generalized characters (for example, as a fusiform actinopterygian) and attribution of specimens to ‘waste-basket’ genera, which masks true diversity. Six Palaeozoic actinopterygian genera (*Acrolepis*, *Amblypterus*, *Elonichthys*, *Palaeoniscum*, *Platysomus* and *Rhadinichthys*) exhibit key characteristics of ‘waste-baskets' [9,78]. They comprise significant proportions of occurrences (averaging more than 30% of occurrences in post-Devonian intervals: electronic supplementary material, figure S10) and occur in contemporaneous freshwater and marine deposits (electronic supplementary material, figure S1), providing further indication that they represent multiple taxa. These ‘waste-baskets' have the highest mean rank in occurrence distributions (electronic supplementary material, figure S11) and serve to concentrate frequency counts of the most common genera, contributing to unevenness in the occurrence distribution and distortion of coverage-based rarefaction estimates [22,23]. The intervals most heavily biased towards frequent taxa (which also tend to contain ‘waste-basket’ taxa; electronic supplementary material, figure S11) are the late Visean (*Elonichthys*: 54/266 occurrences; *Rhadinichthys*: 54/266 occurrences), Kasimovian and Gzhelian (*Elonichthys*: 65/230; *Sphaerolepis*: 60/230), Asselian and Sakmarian (*Paramblypterus*: 53/154 occurrences; *Amblypterus*: 30/154 occurrences), and Wuchiapingian and Changhsingian (*Palaeoniscum*: 66/225 occurrences; *Platysomus*: 26/225 occurrences). Compounding the issue caused by high frequency is the fact that these intervals tend to also have very long-tailed occurrence distributions, with many doubletons or singletons (electronic supplementary material, figure S7). One explanation for these long-tails is the fact that the literature is biased towards descriptions of new taxa rather than occurrences of already-known taxa [45], resulting in an increase of doubletons and singletons. Given that the rate at which new Palaeozoic actinopterygians are being described continues to rise [9], it is unlikely that this trend will change (also meaning that rarefaction curves will not asymptote soon, causing problems for diversity estimation; electronic supplementary material, figure S4).

In the late Carboniferous and early Permian, however, high frequency of taxa results from a combination of ‘waste-basket’ occurrences, intensive sampling and potentially palaeoenvironment, with many separate freshwater localities from Central European deposits yielding thousands of specimens of common genera (e.g. *Elonichthys*, *Sphaerolepis* and *Paramblypterus* [79,80]). Diversity in these intervals is principally derived from fluvio-lacustrine deposits designated as Lagerstätten [5]. As fossil fish tend to be better preserved in freshwater environments than marine [81], this may translate to easier identification of taxa in freshwater environments, potentially biasing richness measures. While there is no relationship between palaeoenvironment and rank occurrence in the Palaeozoic overall (electronic supplementary material, figure S1), the intervals with the highest frequencies of taxa are those that heavily sample freshwater deposits. Whether this phenomenon principally derives from a taphonomic bias or intensive research effort [79,80,82–84] is difficult to ascertain. As coverage-based rarefaction produces lower estimates when evenness is low [23], these highly uneven intervals have low richness estimates at lower quorum levels (figure 3; electronic supplementary material, figures S3–S5). By contrast, at high quorums, where more taxa in the occurrence distribution can be sampled, uneven intervals receive much higher richness estimates (see exponential rise in the rarefaction curves of uneven intervals at high coverage; electronic supplementary material, figures S4 and S5). These large changes at higher quorums in uneven intervals, potentially as a result of preservation bias and research effort, point towards a significant impact of taphonomic filters and anthropogenic bias on diversity estimation. Localized investigations of specimen-level occurrences, sampling and rock records, alongside comparison between analogous regions, are necessary to tease apart the variable impact of these biases. In particular, investigating turnover and nestedness (components of beta diversity) will allow for the teasing apart of the impact that uneven sampling can have on evenness from biases caused by taxonomy.

‘Waste-basket’ taxa may also mask true diversity: the dominance of highly frequent taxa means that a high proportion of sampled taxa consists of these few taxa, likely contributing to lower diversity estimates. Revisionary taxonomic work, such as recognizing new genera among previously congeneric actinopterygians [56], and restriction of *Elonichthys* to just three species [57] rather than over 30, will alleviate this issue and mitigate the dominance of highly frequent forms. These revisions, however, have the potential to increase unevenness in the other direction, as new taxa may end up as singletons or doubletons. Concurrently, the oversplitting of taxa, a prevalent issue when taxa are biostratigraphically important (such as in the marine invertebrate fossil record [23]), may be a potential problem where actinopterygians are significant biostratigraphic markers [85–87].

Major variation in evenness between intervals is highlighted in the different trajectories of coverage-based rarefaction curves (electronic supplementary material, figures S8 and S9). Taxonomic and geographical biases are exacerbated by small sample sizes and low coverage, with rarefaction curves crossing multiple times. Higher (more reliable) quorum levels are unobtainable for Palaeozoic actinopterygians due to the high number of singleton taxa (electronic supplementary material, figure S7) controlling Good's *u* [88]. As a result, coverage is generally low (electronic supplementary material, figures S7 and S8) and only low quorums—at which evenness signals are more pronounced [24]—can be used. When evenness varies at low levels of sampling, size-based rarefaction can in fact be less biased than coverage-based rarefaction, especially at low levels of coverage [23]. Trends between coverage- and size-based rarefaction estimates generally agree (electronic supplementary material, figure S12), although size-based rarefaction estimates higher diversity in some highly uneven intervals (e.g. late Visean; Wuchiapingian and Changhsingian). Small sample sizes (less than 200 occurrences) also have an effect on the accuracy of coverage estimates using Good's *u* [23]: only four of the sampled Palaeozoic intervals have more than 200 occurrences (late Visean: 266; Serpukhovian: 204; Kasimovian and Gzhelian: 230; Wuchiapingian and Changhsingian: 232). Coverage-based rarefaction curves (electronic supplementary material, figures S4 and S5) show these intervals to have among the highest coverage, along with the Bashkirian and Moscovian, highlighting the greater sampling of the Carboniferous than the Permian. Consequently, variation in evenness between intervals is having an overriding effect on sampling-standardized diversity estimates through time, with diversity estimates mostly tracking evenness and reflecting biases in the underlying data [23,89].

## 5. Conclusion and future directions

We present here the first local richness and palaeogeographic trends in Palaeozoic ray-finned fishes. Sampling of the Palaeozoic actinopterygian fossil record is heavily biased towards western Europe (especially the UK) and North America, translating to a very restricted palaeogeographic spread for most of the Palaeozoic. A suite of compounding problems plagues the actinopterygian fossil record and results in bias towards occurrences of both highly frequent and singleton taxa, variation and unevenness in and between sampled intervals, and distortion of relative richness estimates. This confounds attempts to accurately estimate relative richness between intervals. Meanwhile, sampling is poor for regions other than Europe and North America for all but a few Carboniferous and Permian intervals, resulting in an inability to generate meaningful diversity estimates.

Identifying the underlying issues with Palaeozoic actinopterygian data and the interweaving biases that are impacting the fossil record is crucial, and improving sample sizes and coverage will help to mitigate the sensitivity to evenness [25]. Documenting and including existing ‘dark data’ [58,59] in museum collections, as well as focus on new material from undersampled regions, represent key first steps. More complete sampling of well-known regions [90] may facilitate the deduction of accurate local richness patterns [43]. This strategy also goes some way towards accounting for the significant spatial structuring of ‘global’ fossil records [17–19,29]. Spatial standardization, perhaps by restricting analyses to well-sampled localized regions spanning shorter intervals for which specimen- and outcrop-level data is available, represents a key avenue for research. Additionally, examining hierarchical patterns of diversity and patterns of beta diversity [91] will allow for a detailed investigation of the interwoven effects of uneven sampling, taxonomic bias and taphonomy.

Other recently proposed methods such as extrapolation of coverage-rarefied samples [23,24] represent prospective avenues of research, both at local and global scales. However, existing global occurrence data for Palaeozoic actinopterygians is as yet inadequate for such combinations of extrapolation and rarefaction: sample sizes vary widely between intervals, which may result in inaccurate extrapolated richness trends [23,92,93]; sample sizes in all intervals are too low for size-based rarefaction curves to asymptote (electronic supplementary material, figure S4), meaning sample size has an overwhelming effect on diversity estimates [23]; and occurrence distributions are also highly uneven, which biases extrapolators (though to a lesser extent than coverage-based rarefaction [17]).

Overall, the occurrence data recorded in the literature is heavily impacted by sampling and probably results in inaccurate estimated and reported diversity trends at present. Differentiation of the freshwater and marine fossil records and localized diversity estimates for well-sampled regions present feasible avenues of research for reconstructing regional diversity. In addition, research efforts to fix problematic taxonomy of ‘waste-basket’ taxa, in hand with a general increase in sampling, open the possibility of estimating diversity in a spatially standardized framework, so that we can truly begin to understand the origin, rise and establishment of the largest vertebrate clade.

## Data Availability

Code to run the analyses and additional data are provided in the electronic supplementary material [94].
